# Design of a Nanowatt-Level-Power-Consumption, High-Sensitivity Wake-Up Receiver for Wireless Sensor Networks

**DOI:** 10.3390/mi17020178

**Published:** 2026-01-28

**Authors:** Yabin An, Xinkai Zhen, Xiaoming Li, Yining Hu, Hao Yang, Yiqi Zhuang

**Affiliations:** School of Microelectronics, Xidian University, Xi’an 710126, China

**Keywords:** clock synchronization, IOT, low-power wake-up receiver, RFID, WSN

## Abstract

This paper addresses the core conflict between long-range communication and ultra-low power requirements in sensing nodes for Wireless Sensor Networks (WSNs) by proposing a wake-up receiver (WuRx) design featuring nanowatt-level power consumption and high sensitivity. Conventional architectures are plagued by low energy efficiency, poor demodulation reliability, and insufficient clock synchronization accuracy, which hinders their practical application in real-world scenarios like WSNs. The proposed design employs an event-triggered mechanism, where a continuously operating, low-power WuRx monitors the channel and activates the main system only after validating a legitimate command, thereby significantly reducing standby power. At the system design level, a key innovation is direct conjugate matching between the antenna and a multi-stage rectifier, replacing the traditional 50 Ohm interface, which substantially improves energy transmission efficiency. Furthermore, a mean-detection demodulation circuit is introduced to dynamically generate an adaptive reference level, effectively overcoming the challenge of discriminating shallow modulation caused by signal saturation in the near-field region. At the baseband processing level, a configurable fault-tolerant correlator logic and a data-edge-triggered clock synchronization circuit are designed, combined with oversampling techniques to suppress clock drift and enhance the reliability of long data packet reception. Fabricated in a TSMC 0.18 µm CMOS process, the receiver features an ultra-low power consumption of 305 nW at 0.5 V and a high sensitivity of −47 dBm, enabling a communication range of up to 400 m in the 920–925 MHz band. Through synergistic innovation at both the circuit and system levels, this research provides a high-efficiency, high-reliability wake-up solution for long-range WSN nodes, effectively promoting the large-scale application of WSN technology in practical deployments.

## 1. Introduction

**Figure 1:**
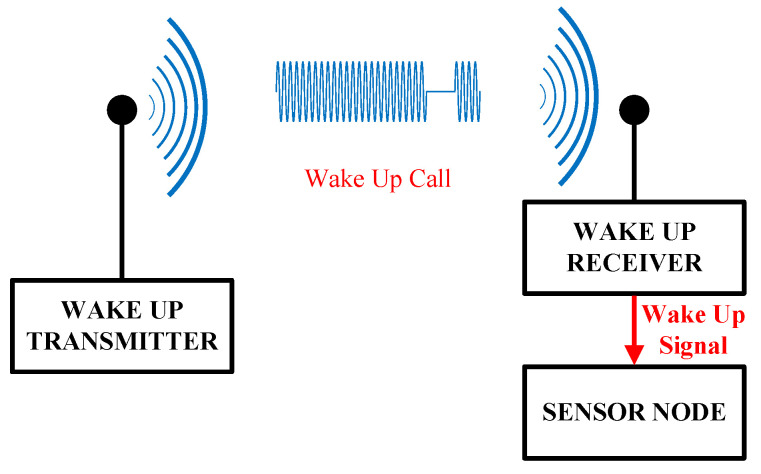
Wake-up receiver concept: the sensor node wakes up only when the correct wake-up call is detected.

With the evolution of Internet of Things (IoT) technology and its widespread deployment in wireless sensor networks [1], the contradiction between the limited energy capacity of battery-powered sensing nodes and the demand for long-term unattended operation has become increasingly prominent, posing a core challenge that restricts their practical application [2,3]. Among various sources of power consumption, the standby power consumed by nodes in the idle listening state is a primary factor limiting their operational lifetime. To address this issue, wake-up receiver technology has been proposed as an effective low-power solution. This technique significantly reduces the overall standby power consumption of the system by incorporating an ultra-low-power auxiliary wake-up receiver [4,5], which operates independently from the main transceiver and remains in a continuous listening state. This auxiliary receiver activates the high-power main system for communication, which handles high-speed data transmission tasks only upon validating a legitimate wake-up command [6,7]; the operating principle of the system is illustrated in Figure 1. This mechanism not only substantially extends the node’s operational duration but also helps improve the system’s communication range as the main receiver can operate in a higher-performance mode. Consequently, researching wake-up receivers that combine high sensitivity with ultra-low power consumption is crucial for promoting the large-scale practical deployment of IoT technology [8].

**Figure 2:**
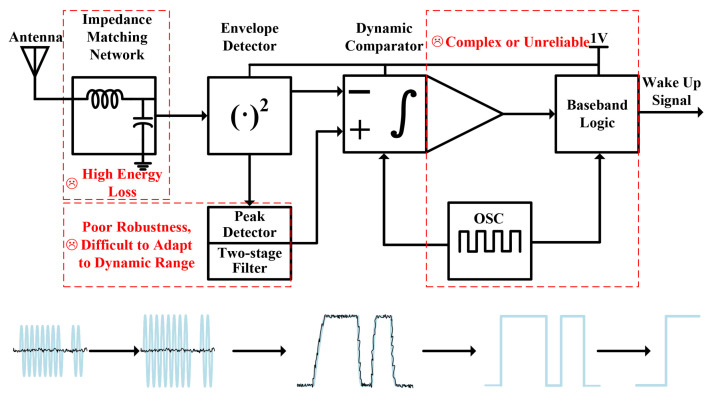
Conventional Wake-up Receiver Architecture.

To achieve low power consumption and long-range communication, semi-active IoT nodes often adopt a typical wake-up receiver architecture, as illustrated in Figure 2 [9]. Operating from a 1-V supply voltage, the signal processing flow of this system is as follows: The RF signal captured by the antenna is first amplified in voltage through a high-Q passive impedance transformation network. Subsequently, its DC component is extracted by an envelope detector. This DC signal is synchronized with a clock provided by an oscillator and fed into a decision circuit composed of a dynamic comparator. The reference level for the comparator is generated by a two-stage average filtering circuit to suppress interference. The recovered baseband digital signal is then sent to a digital correlator for matching verification against a pre-stored wake-up code. A wake-up signal is generated to activate the main receiver if the match meets predefined fault-tolerance criteria.

Although this conventional architecture has achieved certain results in balancing power consumption and performance, it still faces several fundamental technical challenges in practical deployment that hinder its large-scale commercial adoption. First, in terms of system energy efficiency, the widespread use of standard 50 Ohm interfaces and discrete component matching networks introduces significant insertion losses that inherently limit energy transmission efficiency. This creates a trade-off between communication distance and system power consumption [10,11]. Furthermore, when operating in low-frequency bands, the requisite antenna size for effective electromagnetic wave radiation is often large, which is detrimental to the miniaturization and integrated design of IoT node devices. Second, regarding demodulation reliability, when a node is located in the near-field region, signal saturation can easily cause a sharp decrease in modulation depth. Under such shallow modulation conditions, the discrimination capability of traditional peak-detection circuits degrades significantly due to the lack of an effective adaptive decision mechanism. Third, at the level of clock synchronization and system implementation, sampling systems based on low-precision local oscillators suffer from accumulating phase errors due to clock frequency drift. This phenomenon worsens with increasing sampled data length, severely impacting the reliability of long data packet reception, while existing architectures lack effective clock synchronization mechanisms to compensate for such errors. Although employing high-frequency external crystal oscillators or FPGA-assisted processing can improve timing precision and interference immunity, these solutions typically introduce additional power consumption and cost. This contradicts the core requirements of IoT terminals for low cost, miniaturization, and low power consumption, thereby greatly limiting their potential for large-scale application in practical scenarios [12,13]. The above analysis indicates that conventional wake-up receiver architectures exhibit significant shortcomings in energy efficiency, demodulation robustness, clock synchronization accuracy, and system integration. Therefore, coordinated innovation at both the circuit and system levels to develop low-power design solutions with high sensitivity, high reliability, and ease of integration is a key research direction for advancing this technology toward practical applications.

The proposed wake-up receiver employs a high-Q rectifier circuit based on antenna-rectifier co-design in its RF front-end to perform envelope detection. Compared to the conventional approach utilizing a standard 50 Ohm antenna with an LC matching network, this design achieves 2 dB lower insertion loss and higher sensitivity. Moreover, the customized antenna offers greater design flexibility, facilitating better impedance matching under high-Q resonant conditions. A Barker code sequence is adopted as the wake-up preamble, providing enhanced robustness against both interference and noise. Furthermore, leveraging a low-power on-chip clock, an innovative edge-triggered synchronous reset clock circuit is proposed, which more than doubles the tolerance to on-chip clock frequency deviation.

## 2. System Architecture

The RF front-end circuit utilizes an antenna-rectifier co-design methodology to realize passive RF envelope detection with high quality factor (Q) and high sensitivity. The designed front-end achieves a Q-factor of 45. By incorporating a baseband amplifier and an averaging detection circuit, the receiver attains a sensitivity better than −50 dBm while maintaining correct demodulation capability for modulated signals with a modulation depth of 30%. This co-design approach achieves conjugate impedance matching between a custom-designed antenna and the chip, thereby eliminating the LC matching network required in conventional architectures and enhancing the system’s wake-up sensitivity. The input impedance of the proposed RF front-end after Chip-on-Board (COB) packaging is 6 − j280 Ohm. In contrast, if a conventional design employing a standard 50 Ohm antenna with an LC matching network were used—typically selecting an inductor of 47 nH—the surface-mount inductor would exhibit a Q-factor in the range of 30 to 50 at 922.5 MHz, introducing an insertion loss of 1.5 to 2 dB. Such loss is generally unacceptable in high-sensitivity applications. Furthermore, impedance transformation networks in high-Q systems present practical matching challenges, often resulting in degraded S11 performance.

Furthermore, depending on the application scenario, the customized antenna can be flexibly designed according to the specific input impedance of the chip, as well as integration and size requirements, thereby enabling higher system flexibility.

The auto-correlation circuit utilizes a 7-bit Barker code as the wake-up preamble. Moreover, the proposed on-chip edge-triggered synchronous reset clock circuit can clear the accumulated phase of the sampling clock at the data edges. Compared to a non-synchronous reset clock, this design enhances the tolerance to sampling clock frequency deviation from ±7.1% to ±16.7%.

The sensitivity of the proposed wake-up receiver primarily hinges on the sensitivity of the passive RF front-end rectifier. Specifically, when the input power drops below approximately −50 dBm, the voltage amplitude at the antenna port is merely on the order of 10–20 mV. Under such conditions, the MOS transistors operate in the deep subthreshold region, approaching the cut-off state, which results in an extremely high equivalent output resistance of the rectifier and virtually negligible drive capability. The rectifier output voltage, being only a few millivolts, becomes highly susceptible to being overwhelmed by noise or interference in its high-impedance state. After amplification, the signal-to-noise ratio (SNR) of the envelope-detected baseband signal is significantly enhanced, rendering the impact of thermal noise on the amplified baseband signal essentially negligible. Furthermore, to ensure the reliability of the demodulation circuit, the minimum input swing of the averaging detection circuit is set to 60 mV, thereby making it similarly robust against noise interference. Additionally, in the wake-up logic section, the Barker-code autocorrelation circuit also exhibits strong anti-interference capability. Consequently, the trade-off between system receiving sensitivity and robustness is mainly embodied in the antenna-rectifier co-design. The proposed high-Q antenna-rectifier resonant system delivers substantial passive gain; however, it also leads to narrowed system bandwidth and makes the resonant frequency susceptible to environmental variations, which can consequently degrade sensitivity. Therefore, after several design iterations, the rectifier Q-factor is chosen to be around 40 to strike a balance between system sensitivity and robustness.

**Figure 3:**
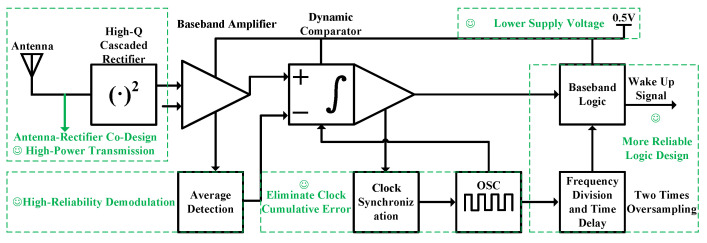
Improved Wake-up Receiver Circuit Architecture.

The improved wake-up receiver circuit architecture designed in this work is illustrated in Figure 3. In standby state, only the wake-up receiver module remains active, with its quiescent current rigorously maintained at an extremely low level. When a reader initiates communication, it first transmits a wake-up signal encoded with a specific sequence, such as a 7-bit Barker code. The RF signal captured by the tag antenna undergoes envelope detection via a high-Q multi-stage cascaded rectifier structure. This structure employs a direct conjugate matching design between the antenna and the rectifier, replacing the traditional 50 Ohm interface and discrete matching network, which significantly reduces the insertion loss of the passive matching network and enhances the RF-to-DC energy conversion efficiency, thereby laying the foundation for high-sensitivity reception. The detected envelope signal is then fed into a mean-detection demodulation circuit. This circuit dynamically generates the reference level for the decision comparator by tracking the peak and valley values of the input signal in real time, thereby overcoming the failure of traditional peak detection in discriminating shallow modulation caused by signal saturation in the near-field region, and enhancing the dynamic range and robustness of demodulation. The demodulated baseband data is subsequently verified by a low-power correlator. A reliable wake-up command to activate the main receiver is generated by calculating the Hamming distance [14] between the received sequence and the preset wake-up code, incorporating a configurable fault-tolerant threshold. To ensure reliable sampling of long codes under ultra-low power constraints, the system integrates data-edge-triggered clock calibration logic, which resets the internal oscillator at each data transition edge, effectively suppressing cumulative sampling errors induced by clock drift. The proposed design achieves co-optimization across the system architecture, RF front-end, analog demodulation, and digital baseband, significantly improving the sensitivity and reliability of the wake-up receiver while maintaining nanowatt-level ultra-low power consumption, thus providing an effective low-power solution for long-range Internet of Things (IoT) communication.

## 3. Circuit Design and Simulation

### 3.1. Co-Design of Rectifier and Loop Antenna for High-Sensitivity Demodulation Systems

**Figure 4:**
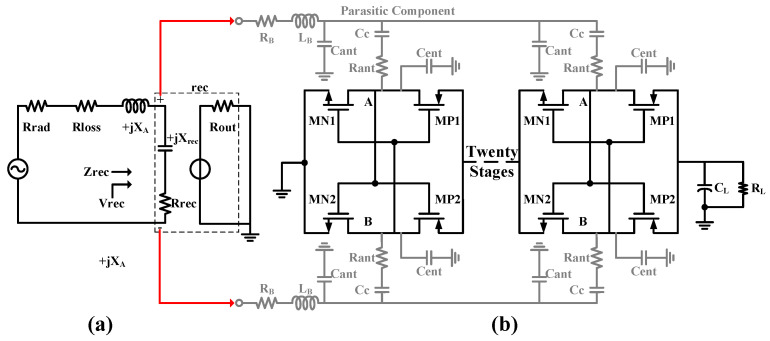
(**a**) Antenna-Rectifier Equivalent Model. (**b**) Threshold Voltage Self-Canceling Rectifier.

To meet the stringent requirements for high-sensitivity signal demodulation in low-power wake-up receivers, this section presents a co-design methodology for a rectifier and a loop antenna. This approach breaks from the conventional 50 Ohm matching network constraint by achieving direct conjugate impedance matching between the antenna and the multi-stage rectifier, with the goal of maximizing the energy transfer efficiency in the RF front-end [15]. Figure 4a illustrates the equivalent circuit model of the proposed co-design. In this model, the antenna is characterized as a Thevenin equivalent circuit comprising a radiation resistance ($R_{rad}$), a loss resistance ($R_{loss}$), and an inductive reactance ($+jX_{A}$). The input impedance of the rectifier exhibits a capacitive characteristic ($-jX_{rec}$). The quality factor (Q) of the rectifier is calculated as follows:

(1) $$Q=\frac{X_{S}}{R_{S}}=\frac{R_{P}}{X_{P}}$$

The equivalent parallel resistance and reactance are given by

(2) $$R_{P}=\left(1+Q^{2}\right)R_{S}$$

(3) $$X_{P}=\left(1+\frac{1}{Q^{2}}\right)X_{S}$$

The available output power at the antenna port is

(4) $$P_{av}=\frac{{\left(\frac{V_{L}}{\sqrt{2}}\right)}^{2}}{R_{S}\left(1+Q^{2}\right)}=\frac{{\left(\frac{V_{L}}{\sqrt{2}}\right)}^{2}}{R_{S}+\frac{X_{S}^{2}}{R_{S}}}\approx \frac{R_{S}{\left(\frac{V_{L}}{\sqrt{2}}\right)}^{2}}{X_{S}^{2}}$$

High-sensitivity design aims to minimize the available power delivered by the antenna port for a given output voltage amplitude. From the above equations, it follows that higher sensitivity requires a higher quality factor (Q) of the rectifier and, simultaneously, a smaller real part of the impedance. The passive gain of the antenna is expressed as

(5) $$G_{V,\mathrm{boost}}=\left|\frac{V_{\mathrm{rec}}}{V_{A}}\right|=\frac{R_{\mathrm{rec}}+1/\left(j\omega C_{\mathrm{rec}}\right)}{R_{A}+R_{\mathrm{rec}}}\approx \frac{X_{\mathrm{rec}}}{R_{A}+R_{\mathrm{rec}}}$$

where Va is the amplitude of the antenna equivalent voltage source and Vrect is the output voltage at the antenna port. Hence, the passive gain of the antenna rectifier LC resonant tank is Q/2, which further demonstrates that a high sensitivity rectification system necessitates a high Q design. However, a higher Q is not always preferable, as it increases the difficulty of antenna design and impedance matching. In this work, the design value of Q is chosen to be in the range of 30 to 50.

The rectifier employs a differentially driven cross-coupled topology. The advantage of this structure is that the cross-coupled connection dynamically compensates for the MOSFET threshold voltage, thereby reducing the effective turn-on voltage of the transistors. Simultaneously, transistors with a medium threshold voltage, approximately 300 mV, are used. Under conditions of low input power, the voltage induced at the antenna port is too low, forcing the transistors to operate in the deep sub-threshold region. A multi-stage cascaded configuration is therefore essential to boost the output voltage. To minimize parasitic capacitance, the transistor channel length L is set to its minimum value. In the design iteration, the number of stages primarily depends on the antenna port voltage and the target output voltage. The output voltage itself is determined by the design threshold of the subsequent circuitry. In this work, to enhance system robustness, the output voltage must not fall below 20 mV. The determination of the other two key design variables—the number of stages and the transistor width W—embodies the core concept of the antenna-rectifier co-design. The iterative design flow is as follows:

Step 1: Given a rectifier input port voltage (e.g., 10 mV), initial values are set for the number of stages and W. The output voltage, impedance, and Q-factor are then simulated.

Step 2: Based on the results from Step 1, a matching antenna is designed. Its gain, efficiency, and size are obtained, and the system sensitivity is evaluated.

Step 3: The process loops back to Step 1. The number of stages and W are iteratively optimized, with corresponding antennas designed and system sensitivity calculated for each iteration.

Step 4: After multiple optimization iterations, the system sensitivity converges to an optimal range, indicating that the system performance has reached its best balance point.

Upon completion of the iterative design, the rectifier employs a 20-stage cascade. When the antenna port voltage is 10 mV, the output exceeds 20 mV. The final rectifier design achieves a Q-factor of 40. After impedance matching, the sensitivity is better than −50 dBm.

In the practical design process, the impact of the layout on performance is critical. Given that the rectifier’s input impedance is primarily constrained by its internal parasitic capacitances, and considering that pre-layout simulation results may deviate significantly from post-layout simulations incorporating parasitic parameters—while rectification sensitivity is directly dependent on the accuracy of the input impedance—a specific design flow was adopted. This flow involved first completing the layout of the cascaded rectifier, followed by parasitic parameter extraction for post-layout simulation. Finally, the extracted parasitic parameters were back-annotated to the schematic for re-simulation, thereby minimizing impedance deviation.

**Figure 5:**
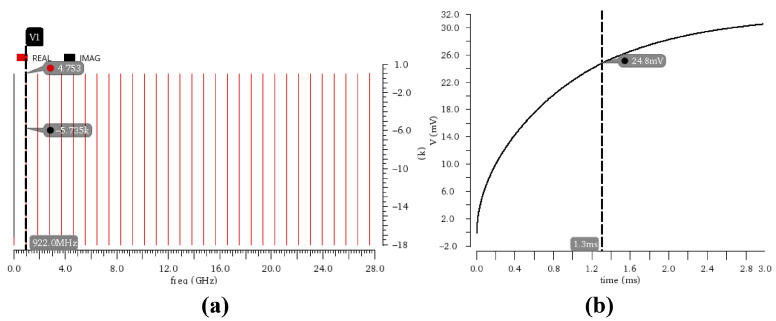
Simulation Results of the 20-Stage Cascaded Rectifier: (**a**) Input Impedance PSS Simulation. (**b**) Transient Response Characteristics.

Simulation results (Figure 5a) demonstrate that through precise optimization of the NMOS transistor width-to-length ratio (W/L), the rectifier achieves an input impedance of 4 − j×372 Ohm at the target operating frequency of 922.5 MHz. This provides a foundation for effective co-design with the antenna. Further simulations (Figure 5b) verify that when the input signal amplitude is as low as 10 mV, the structure, leveraging its multi-stage voltage multiplication effect, can generate an output voltage exceeding 25 mV within 1.3 ms. This output is sufficient to reliably trigger subsequent circuitry into operation.

**Figure 6:**
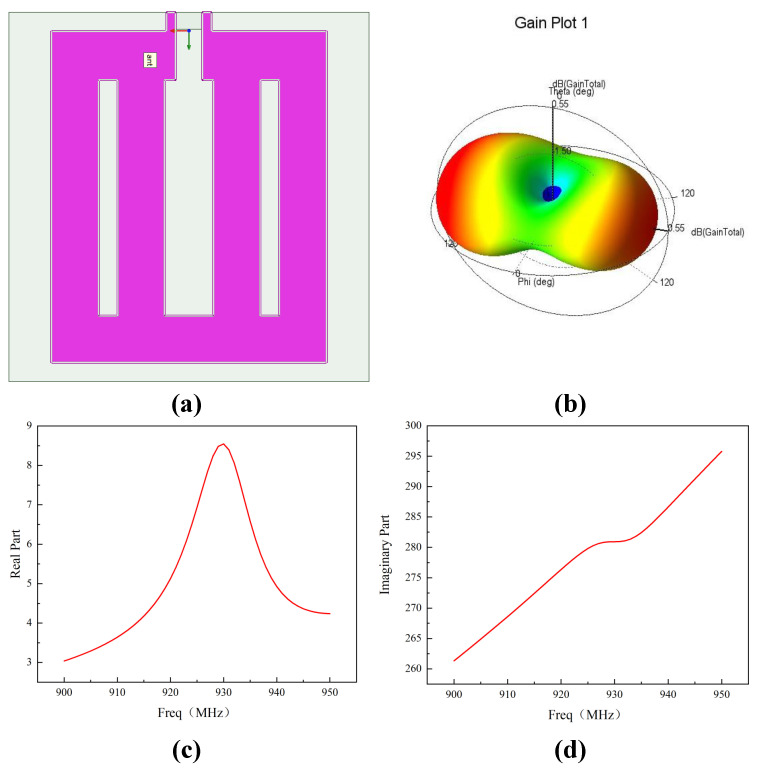
(**a**) Antenna Structure. (**b**) Simulated Gain Pattern. (**c**) Real Part of Input Impedance. (**d**) Imaginary Part of Input Impedance.

At 922.5 MHz, a dipole antenna would have a size approximating half the wavelength of the electromagnetic wave, exceeding 15 cm. This dimension is considered too large to meet the compact-device design requirements of the wake-up receiver. To achieve a balance between compact size and performance, this paper adopts a loop antenna topology. The design of the customized antenna is illustrated in Figure 6. Accounting for post-layout simulation results of the chip and the parasitic capacitance introduced by the Chip-on-Board (COB) packaging, the antenna impedance is designed to be 5 + j×280 Ohm. The antenna measures 4.5 cm by 4.8 cm, with a gain of 0.76 dBi. This design prioritizes a compact form factor at the expense of higher gain, achieving a radiation efficiency of 82%. The radiation efficiency is calculated using the following formula:

(6) $$\eta_{\mathrm{rad}}=\frac{R_{\mathrm{rad}}}{R_{\mathrm{loss}}+R_{\mathrm{rad}}}$$

The calculated Rloss resistance is approximately 0.8 Ohm. Due to the high Q-factor of the rectifier and the frequency-dependent variations in both the real and imaginary parts of the antenna impedance, the system exhibits a relatively narrow bandwidth. Simulation results indicate a 3-dB bandwidth of 20 to 25 MHz. Despite the sensitivity of antenna impedance to fabrication and environmental conditions, the loop antenna used in this design exhibits a gradual frequency-dependent impedance variation. This characteristic allows the high-Q resonant system to retain sufficient matching bandwidth under practical operating conditions. Figure 6 depicts the key characteristics of the proposed antenna: (a) the antenna structure, (b) the simulated antenna gain, (c) the evolution of the real part of the antenna impedance versus frequency, and (d) the evolution of the imaginary part of the antenna impedance versus frequency.

### 3.2. Mean-Detection Demodulation Circuit Design for Shallow Modulation Applications

#### 3.2.1. Signal Amplifier

To meet the performance requirement of high-sensitivity reception in long-range communication scenarios, this paper designs a dedicated signal amplification circuit for the RF analog front-end. When receiving weak RF signals, even after the voltage multiplication effect of the rectifier, the amplitude of its output signal typically remains at the millivolt level, which is insufficient to effectively drive the subsequent decision circuit. Therefore, introducing a signal amplification stage with substantial gain is crucial. This stage must amplify the DC signal from the rectifier output to a level suitable for reliable decision-making by the subsequent comparator.

In an architecture that employs passive gain and prioritizes envelope detection, and where no RF amplification stage is placed at the very front-end of the signal path, the overall sensitivity of the system largely depends on the noise introduced by the envelope detector and the subsequent amplification circuit. Consequently, careful noise optimization of the amplifier circuit becomes a key design consideration for achieving high sensitivity [16]. Simultaneously, given the system’s stringent requirement for ultra-low power consumption, the amplifier circuit must achieve excellent noise performance within a limited power budget. Based on this, instead of adopting complex, high-power-consumption special circuit structures, this design opts for a standard low-power architecture followed by targeted optimization and design.

**Figure 7:**
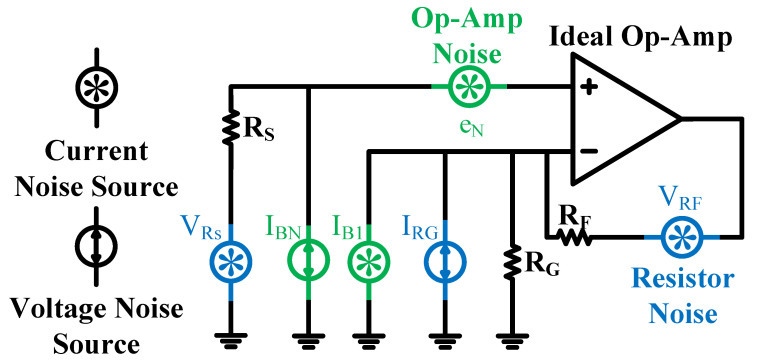
Equivalent Circuit of the Amplifier’s Noise Sources.

Figure 7 shows the equivalent circuit model of the non-inverting operational amplifier, including its noise sources. The total output noise of this circuit is primarily composed of the root mean square sum of individual noise components. Its theoretical expression is given by

(7) $$e_{o}=\sqrt{\left(4KTR_{S}+{\left(I_{BN}R_{S}\right)}^{2}+e_{N}^{2}\right){\left(1+\frac{R_{F}}{R_{G}}\right)}^{2}+{\left(I_{B1}R_{F}\right)}^{2}+4KTR_{F}\left(1+\frac{R_{F}}{R_{G}}\right)}$$

Noise analysis was performed and verified using the Cadence simulation platform (version IC618). Owing to the narrow bandwidth of the amplifier, the equivalent input-referred noise voltage, calculated by integrating the internal noise over the frequency band of interest and referring it to the input, is approximately 41.2 µV. This noise level is within the acceptable design tolerance and meets the specified noise performance requirements for the front-end amplification circuit.

#### 3.2.2. Mean-Detection Circuit

In conventional design schemes, the reference voltage for the decision comparator is typically generated by either a low-pass filter or a peak-detection circuit. When employing a low-pass filter, the essence of the approach lies in extracting the signal average using an RC filtering network with a large time constant. Although the filtering-based method offers good stability, in scenarios where the transmitted signal is a continuous wave, the voltage difference between the filtered output signal and the envelope signal can be minimal, which may easily lead to decision errors. If the peak-detection method is adopted—where the signal peak is detected and held, and half of this value is used as the reference level—this approach heavily relies on the signal’s modulation depth. It requires a deep modulation condition (typically demanding a modulation depth greater than 50%) to function effectively.

**Figure 8:**
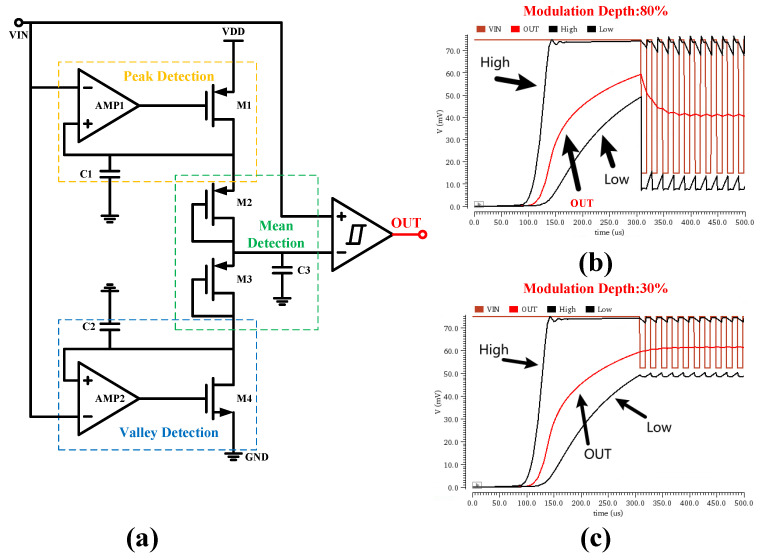
(**a**) Structure of the Mean-Detection Circuit. (**b**) Transient Response Simulation under Deep Modulation. (**c**) Transient Response Simulation under Shallow Modulation.

To address the technical challenges in demodulating shallowly modulated signals, this study proposes an innovative mean-detection circuit architecture, whose basic structure is shown in Figure 8a. This circuit comprises three main parts: a peak detection unit, a valley detection unit, and a voltage divider network. Its core innovation lies in the ability to track the peak (V_max_) and valley (V_min_) values of the input signal in real-time and compute their average to serve as the reference level:

(8) $$V_{\mathrm{ref}}=\frac{V_{\max}+V_{\min}}{2}$$

The peak detection unit (AMP1, MP3, MP4) is responsible for monitoring the input signal. It triggers the corresponding control logic when the input voltage falls below the voltage stored on capacitor C1. The valley detection unit (AMP2, AMP3, MP4), conversely, controls the capacitor discharge process, enabling the voltage to track the signal’s valley level. The voltage divider network (M2, M3, C3) performs level shifting on the processed signal, completing the entire signal conditioning flow. Through precise timing coordination among these units, fast and accurate detection of the input envelope signal is achieved.

To verify the performance of the proposed circuit, simulations were conducted under conditions of 27 °C ambient temperature and a 0.5 V supply voltage. The simulation was set with all devices at the tt process corner. The envelope signal input to the mean-detection circuit was pre-processed by the pre-amplifier, with its DC level set to 75 mV. Simulation results under modulation depths of 80% and 30% are shown in Figure 8b and Figure 8c, respectively. The results demonstrate that the circuit architecture maintains stable performance across different modulation depths, effectively overcoming the limitations of traditional demodulation circuits under shallow modulation conditions.

#### 3.2.3. Dynamic Comparator Design

**Figure 9:**
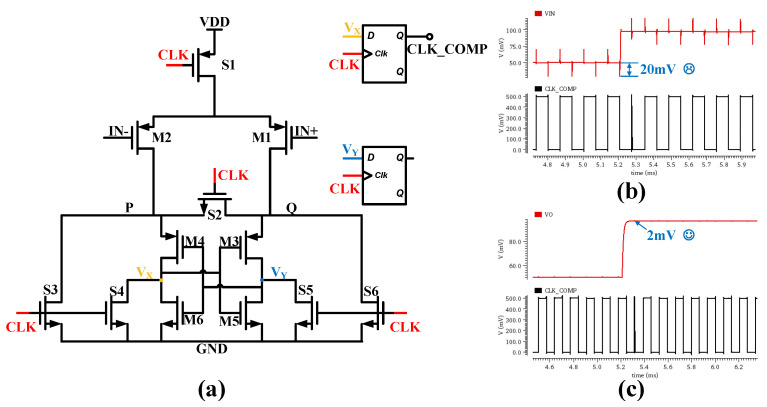
(**a**) StrongArm Dynamic Comparator. (**b**) Kickback Noise in the Dynamic Comparator. (**c**) Input Signal after Kickback Noise Elimination.

To achieve the design goals of low power consumption and high reliability, this study employs a dynamic comparator architecture based on the StrongArm latch topology (its circuit structure is shown in Figure 9a [17], replacing conventional hysteresis comparators that exhibit significant static power consumption. This dynamic comparator operates at a 4 kHz sampling frequency. Its core structure consists of an input differential pair (M1, M2) and a cross-coupled regenerative latch unit (M3–M6), with switching between reset and evaluation phases achieved through seven clock-controlled switches (S1–S6).

When the clock signal (CLK) is high, the circuit enters the reset phase, and its internal critical nodes are reset to ground (GND). When the clock signal transitions to low, the circuit enters the evaluation phase: the input pair transistors M1 and M2 turn on, allowing the supply voltage (VDD) to charge the nodal capacitors through them. An input voltage difference $\left(\Delta v_{in}\right)$ causes a current imbalance between the left and right branches. This current difference is rapidly regenerated and amplified by the cross-coupled positive feedback latch structure formed by M3–M6, driving the node with the higher voltage quickly to the logic high level and the other node to the logic low level. The output signal is conditioned by a buffer stage and then precisely latched by a subsequent D-type flip-flop on the rising clock edge. To eliminate offset introduced by load mismatch, a symmetrical D-type flip-flop structure is also configured in the other differential signal path. One of the primary technical challenges in dynamic comparators is kickback noise. This noise originates from voltage spikes coupled into the preceding sensitive circuitry through parasitic parameters—such as gate-to-source capacitance (Cgs), body-to-source capacitance (Cbs), and gate-to-drain capacitance (Cgd)—of switching transistors (M1 and M2) when the clock signal transitions.To effectively suppress kickback noise, this study introduces a filtering capacitor at the input stage of the comparator. The circuit employs a 1 pF capacitor, whose value is significantly larger than the parasitic capacitances of the switching transistors. As shown in the VIN signal in Figure 9b—which corresponds to the case without the filter capacitor at the comparator input—a noise glitch of approximately 20 mV caused by kickback noise is clearly observable. Such glitches can readily lead to comparison errors in subsequent cycles. In contrast, as illustrated in Figure 9c, after adding the filter capacitor at the comparator input, the kickback noise on the VO signal is effectively suppressed. The resulting kickback voltage at the input manifests only as a minor pulse spike of about 2 mV, which does not cause erroneous decisions.

#### 3.2.4. Performance Simulation and Verification

System-level simulations were conducted to validate the proposed demodulation circuit operating at a 0.5 V supply voltage. The circuit architecture integrates a fully differential rectifier, a signal amplification circuit, a mean-detection circuit, and a decision comparator.

**Figure 10:**
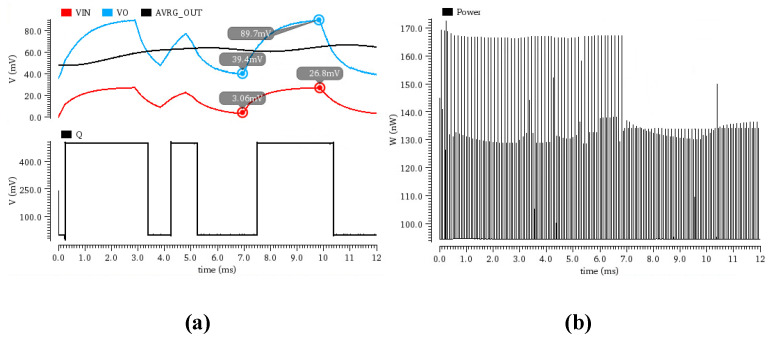
(**a**) Overall Transient Response Simulation of the Demodulation Circuit. (**b**) Overall Power Consumption Simulation of the Demodulator.

Under test conditions of a 25 mV input signal amplitude, a 2 kbps modulation rate, and an 80% modulation depth, the simulation results are shown in Figure 10a. After processing by the amplification circuit, the peak value of the rectifier’s output signal was amplified to 89.7 mV, corresponding to a voltage gain of approximately 3 times. However, the valley value exhibited asymmetric amplification behavior, achieving a gain of 13 times, which consequently reduced the modulation depth from the initial 80% to 56.3%. This phenomenon indicates effective detection capability for weak signals, while also revealing linearity limitations of the amplification circuit under very small input signal conditions, highlighting the necessity for a high dynamic range design. Despite this nonlinear amplification, the demodulated output signal exhibited only a 2% timing error in pulse width, indicating well-maintained timing characteristics. Regarding power consumption, the simulation results, shown in Figure 10b, indicate that the circuit consumes 94.61 nW during normal operation, achieving a good balance between low-power operation and functional integrity. These simulation results provide an important reference for subsequent circuit performance optimization and system-level design.

### 3.3. Correlator Logic Baseband and Clock Synchronization Circuit Design

To address the key challenges in baseband processing and clock management for low-power wake-up receivers, this chapter presents the design and implementation of a high-reliability, low-power correlator logic baseband and clock synchronization circuit. The design employs a wake-up code verification method based on Hamming distance, incorporates a configurable error tolerance threshold, and utilizes a data-edge-triggered synchronization mechanism. Operating stably at an ultra-low supply voltage of 0.5 V, the design effectively mitigates cumulative phase errors and sampling synchronization issues caused by clock drift during long data packet transmission.

#### 3.3.1. Correlator Logic Baseband Design

To meet the stringent ultra-low power requirement of the wake-up receiver, this design adopts a low data-rate encoding scheme, setting the data transmission rate of the analog front-end to 1 kbps. Given the characteristic of the dynamic comparator to maintain its previous sampling state until the arrival of the next sampling clock edge, the sampling frequency should theoretically be maximized to improve decision accuracy. In this scheme, the 8 kHz main clock signal is directly used as the reset clock for the dynamic comparator to achieve oversampling of the input signal. Concurrently, the main clock is frequency-divided and delayed to generate a 2 kHz clock, which serves as the sampling clock for the correlator logic, resulting in two sampling bits per input data bit, thereby effectively implementing oversampling. Research [18,19,20] has demonstrated that oversampling techniques can effectively alleviate phase misalignment caused by clock frequency deviations and possess the potential to suppress noise and enhance the signal-to-noise ratio (SNR).Double oversampling is employed with a 4 kHz clock, thereby further enhancing system robustness. For other application scenarios, the sampling clock frequency needs to be selected according to the specific practical conditions.

**Figure 11:**
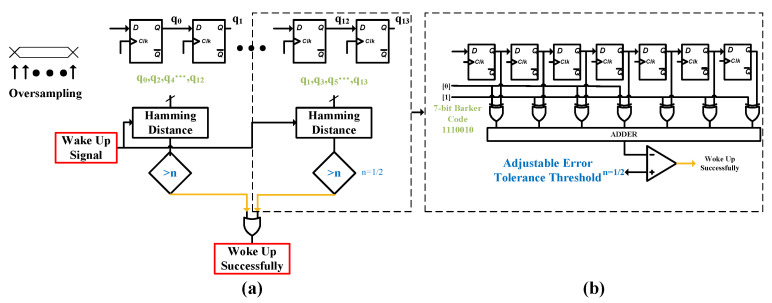
(**a**) Operational Process of the Grouped Correlator. (**b**) Structure Diagram of the 7-bit Correlator Logic.

The serial data output from the comparator is first converted into a parallel format by a shift register before being fed into the subsequent correlator for processing. The data has already been oversampled at the shift register stage, this design adopts a grouped correlator architecture. The primary distinction between this architecture and a conventional correlator lies in its incorporation of two independent operating phases. As illustrated in Figure 11a, each phase consists of a seven-bit shift register with an identical structure, responsible for performing a bit-by-bit comparison between the input bit stream and a locally stored wake-up code. When the correlation value output from either phase exceeds a preset threshold, the system determines that a valid wake-up signal has been detected and generates the corresponding wake-up command.

Figure 11b shows the logic structure of the 7-bit correlator. The correlator logic typically comprises fundamental building blocks such as a shift register, an array of XOR gates, NOT gates, and a multi-bit adder.The shift register, implemented by cascading multiple D-flip-flops, functions to convert serial input data into parallel output. The core operation of the correlator is based on the calculation of the Hamming distance. Each bit of the shift register’s output and the corresponding bit of the preset wake-up code are connected to an XOR gate. When the corresponding bits match, the XOR gate outputs a ‘0’; when they mismatch, it outputs a ‘1’. The outputs of these XOR gates are inverted by NOT gates and then fed into a multi-bit adder for summation. The output of the adder reflects the degree of difference between the received data and the preset wake-up code—specifically, a smaller Hamming distance indicates a higher degree of match, resulting in a larger numerical value at the adder’s output. Finally, this output value is compared against a configurable threshold. If the value exceeds the set threshold, a wake-up signal is generated to activate the main receiver.

By appropriately setting the error tolerance threshold (this system supports 1-bit or 2-bit fault tolerance) [21], a balance can be effectively struck between the probability of false wake-ups and missed wake-ups, thereby enhancing the system’s robustness in practical interference-prone environments. Considering the constraints of coding efficiency, fault tolerance capability, and power consumption collectively, this work selects the 7-bit Barker code (code sequence: 1011100) as the wake-up identifier. Combined with the two-phase oversampling correlator design, this approach significantly improves tolerance to clock frequency deviations, providing a highly reliable wake-up decision solution for low-power Internet of Things (IoT) communication.

#### 3.3.2. Custom Low-Voltage Correlator Logic Using an Analog-Oriented Design Approach

To optimize the overall system power consumption, the supply voltage for this design is set to 0.5 V. However, standard digital cell libraries typically require a 1 V supply, making them unsuitable for direct application in such an ultra-low voltage scenario. This section, therefore, proposes a customized design flow for implementing the low-voltage digital logic and presents simulation results verifying the functionality and power characteristics of the designed circuit. A customized methodology, rooted in analog circuit design practices, is employed for this low-voltage digital logic.

The process begins with a behavioral-level description of the digital baseband module using Verilog HDL. The primary functions of this module include initializing the wake-up command output and resetting the shift register states when the reset signal is asserted, and sampling and shifting the input data on the rising edge of the clock signal to convert serial data into a parallel format. The parallel data bits (specifically bits 0, 2, 4, 6, 8, 10, 12 and bits 1, 3, 5, 7, 9, 11, 13) are grouped into two sets for separate summation operations. A valid wake-up signal is generated if the summation result from either set exceeds a preset fault-tolerant threshold. This threshold is dynamically configurable via an external level signal: a low level indicates the system tolerates a 1-bit error, while a high level indicates tolerance for a 2-bit error.

**Figure 12:**
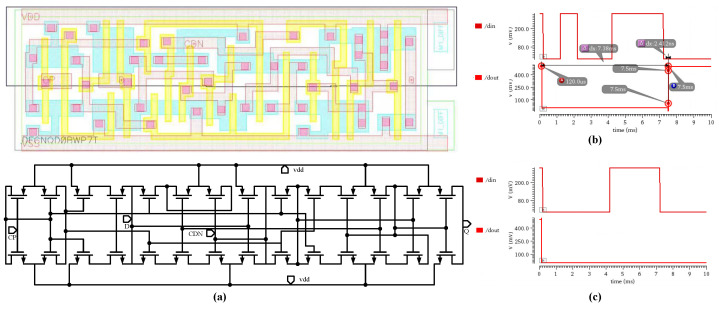
(**a**) Layout and Reverse-Extracted Schematic of the D Flip-Flop. (**b**) Transient Simulation of the Digital Baseband with Correct Operation. (**c**) Transient Simulation of the Digital Baseband with a One-Bit Error in the Wake-up Code.

Following logic synthesis, the generated netlist is imported into the Cadence Virtuoso platform and converted into a circuit schematic. It is important to note that, due to the lack of ready-made low-voltage device schematics for 0.5 V operation in the standard cell library, the circuit structures of basic logic gates were reverse-extracted from the standard cell layouts and manually augmented with power and ground pins. For instance, Figure 12a illustrates the layout structure and the subsequently extracted transistor-level schematic of a low-level reset, positive-edge-triggered D-type flip-flop (DFCNQD0BWP7T).

After finalizing the circuit schematics, a simulation analysis was conducted on the module. The simulation parameters were configured as follows:the operating clock frequency was 2 kHz, the input data rate was 1 kbps, the modulated signal used a 7-bit Barker code (sequence: 0100111) with a high level of 500 mV and a low level of 0 V, and the supply voltage was 0.5 V. The simulation results are shown in Figure 12b. After the system completely received the 7-bit Barker code, it successfully generated a valid wake-up command signal. The output signal’s transition time was 2.4 ns, and the total delay from system reset to the generation of the wake-up command was approximately 7.5 ms, indicating that the wake-up decision was completed at the first rising clock edge after receiving the entire 7-bit code. In terms of power consumption, since the digital baseband module primarily incurs dynamic power consumption during clock transitions, its overall power consumption remains at an extremely low level. Simulation results show that the average power consumption for a complete process, from data reception to wake-up decision, is on the order of picowatts (pW). To evaluate the fault tolerance of the baseband module, a test was further conducted using a Barker code with a one-bit error in the input sequence (sequence: 0000111). As shown in Figure 12c, when the error tolerance threshold was set to zero (i.e., no fault tolerance), the system did not trigger a wake-up command because the input data did not perfectly match the internally preset reference code. This result verifies that the designed baseband module possesses strict false wake-up suppression capability.

#### 3.3.3. Clock Synchronization Circuit Design

In On-Off Keying (OOK) modulated wake-up receivers, the baseband logic must reliably decode the baseband bit stream demodulated by the analog front-end. Existing schemes often employ oversampling or digital control techniques to overcome phase misalignment between the data and the internal clock. When the data rate is fixed, frequency errors primarily stem from deviations of the internal clock source; consequently, a high-precision clock is crucial for system performance [22]. However, conventional high-precision clock sources, such as phase-locked loops (PLLs) or crystal oscillators, are often unsuitable for semi-active, low-power scenarios due to their high power consumption and large area overhead.

The design of the sampling clock must fulfill two key requirements. First, the sampling point should be aligned near the center of the data bit to equally tolerate positive and negative frequency deviations. Second, the accumulation of phase error must be suppressed through a synchronization mechanism to enhance the reliability of long data packet reception, preventing error buildup during sequences of consecutive identical bits. To meet these requirements, this paper proposes a data-edge-triggered clock synchronization logic specifically designed for ultra-low-power and long-data transmission scenarios. Based on a gated oscillator clock and data recovery (GO-CDR) architecture [23], this logic maintains phase and frequency synchronization between the data and the local sampling clock without relying on traditional PLLs.

**Figure 13:**
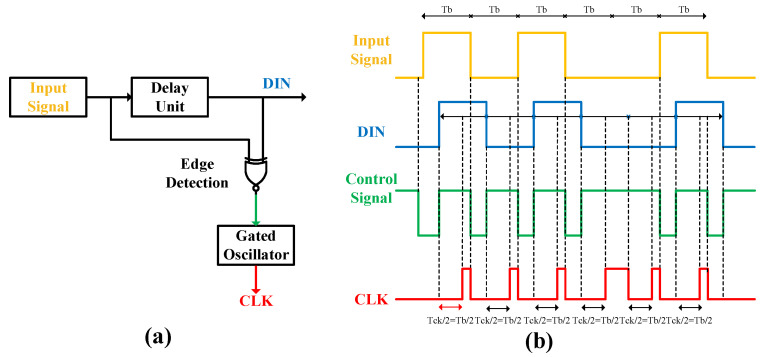
(**a**) Clock Synchronization Logic. (**b**) Signal Timing Diagram of the Clock Synchronization Circuit.

The circuit structure, shown in Figure 13a, primarily consists of three parts: a delay unit, an edge detection circuit based on an XNOR gate, and a gated oscillator. The input data signal is delayed by a unit to generate a DIN signal with a delay of $\tau_{d}$. The edge detector produces a reset pulse with a width of $\tau_{d}$ whenever a transition occurs in the input data; its output remains high at other times. The gated oscillator operates as follows: when its control signal is high, the oscillator runs freely, generating a clock signal with frequency $f_{ck}$ = 1/$T_{ck}$. When a low-level pulse appears on the control signal, the oscillator is reset to its initial state. Assuming the unit interval $T_{b}$ equals the clock period $T_{ck}$, its operational waveform is depicted in Figure 13b.

Each transition in the input data triggers a low-level pulse from the edge detector, which resets the gated oscillator, thereby clearing any accumulated phase error. After the control signal returns high, the oscillator restarts its free-running operation. Ideally, the first rising clock edge occurs at $T_{ck}$/2, aligning the sampling edge to the center of the data bit and achieving synchronization between the data edge and the sampling clock. As long as the control signal remains high, the DIN signal is continuously sampled at the frequency $f_{ck}$. If a discrepancy exists between $T_{b}$ and $T_{ck}$, phase error accumulates during non-transition intervals; however, this error is cleared at the next data transition. Therefore, the limiting factor for long-packet reception is not the total data length but the maximum length of consecutive identical bits (CIB). The sampling circuit is designed to be immune to noisy data edges. In the synchronization logic, a multi-stage inverter delay chain is introduced during the clock synchronous reset to ensure that sampling occurs near the center of the data level. This delay chain is sufficient to cover all data edge noise, thereby guaranteeing that the circuit remains unaffected by noisy data edges when the sampling clock arrives.The circuit proposed in this work achieves a significant power reduction to the nanowatt level while meeting the target application requirements.

#### 3.3.4. Design of the Gated Oscillator, Delay, and Edge Detection Module

**Figure 14:**
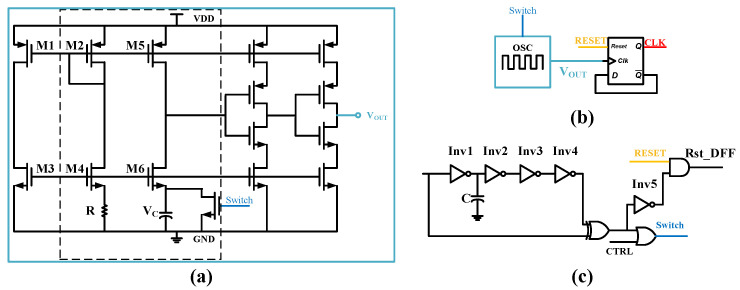
(**a**) Schematic of the Gated Oscillator. (**b**) Clock Signal Holding. (**c**) Structural Diagram of the Delay and Edge Detection Module.

Figure 14a illustrates the gated oscillator circuit proposed in this work [24], which serves as the primary clock source for the wake-up receiver, generating an 8 kHz square wave signal with a 50% duty cycle. This oscillator comprises a reference current generation circuit, an inverter chain, and a reset switch. The reference circuit, where transistors M2 and M4–M6 operate in the subthreshold region, generates a reference current, Iref. By utilizing an external 5.5 M Ohm high-value resistor, Iref is reduced to approximately 7.5 nA, enabling the oscillator to achieve nanowatt-level power consumption. The oscillator operates based on a capacitor charging-discharging mechanism: when the capacitor voltage exceeds a reference voltage, the comparator toggles, producing the clock output. A gating signal, generated by an edge detector, controls the reset switch to discharge the capacitor when necessary for phase synchronization. The output stage employs a current-starved inverter as a buffer to further optimize power consumption.

To enhance clock period stability, a frequency divider circuit based on a D-type flip-flop is incorporated after the oscillator, as shown in Figure 14b.

The delay and edge detection module, depicted in Figure 14c, introduces a controlled delay to the input signal using an inverter chain (Inv1–Inv4) and a nodal capacitor, C. The delayed signal and the original signal are fed into an XOR gate to generate the gating signal. According to the oscillator’s operational logic, the oscillator runs in a free-running state when the gating signal is low. Consequently, this gating signal is OR-combined with the oscillator output, Vout, and the result is used as the reset control signal for the internal charging capacitor of the oscillator. Simultaneously, the inverted gating signal is AND-ed with the global system reset signal, RESET, to generate the reset signal for the subsequent D-type flip-flop (this flip-flop is active low). The delayed signal from the output of the delay module ultimately serves as the baseband data input, which is transmitted to the digital baseband logic unit for subsequent processing.

#### 3.3.5. Overall Simulation of the Clock Synchronization Module

**Figure 15:**
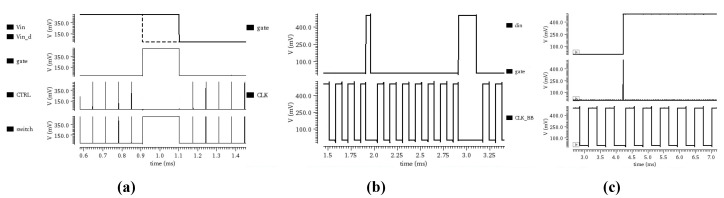
(**a**) Waveforms at Key Nodes of the Delay and Edge Detection Module Simulation, (**b**) Reset Effect of the Capacitor Reset Signal on the Oscillator Output Clock, (**c**) Reset Effect on the Baseband Clock.

To verify the functional correctness and power characteristics of the proposed clock synchronization module, a transient simulation analysis was conducted. The simulation results are shown in Figure 15 The waveform characteristics at key nodes and the corresponding operational mechanisms for each sub-module are elaborated below.

Figure 15a shows the waveforms at the key nodes of the delay and edge detection module. The dashed line represents the input signal (Vin), and the solid line represents its delayed version ($Vin_{d}$) after processing by the delay unit. These two signals are fed into an XOR gate to generate the gating signal (GATE). This gating signal is then OR-ed with the oscillator output signal (CTRL) to ultimately produce the critical signal (switch) that controls the reset of the oscillator’s capacitor.

Figure 15b illustrates the synchronization effect of the capacitor reset signal on the oscillator output clock. When the gating signal is active, the clock signal is reset to zero. After waiting for half a clock cycle, a rising edge is generated, thereby achieving synchronization between the clock signal and the edges of the input data.

Figure 15c demonstrates the reset effect of the gating signal on the baseband clock. Since the baseband clock is derived from the main clock through frequency division and delay, the reset effect of the gating signal is somewhat attenuated. Nevertheless, it remains effective in triggering the synchronization operation, ensuring temporal alignment between the baseband clock and the input signal. The aforementioned simulation results indicate that the proposed clock synchronization circuit can correctly perform data-driven dynamic timing adjustment at an ultra-low supply voltage. It provides an effective solution for accurately positioning sampling points and suppressing cumulative errors caused by clock drift.

## 4. Chip Testing and Result Analysis

### 4.1. Test Solution and Platform Construction

**Figure 16:**
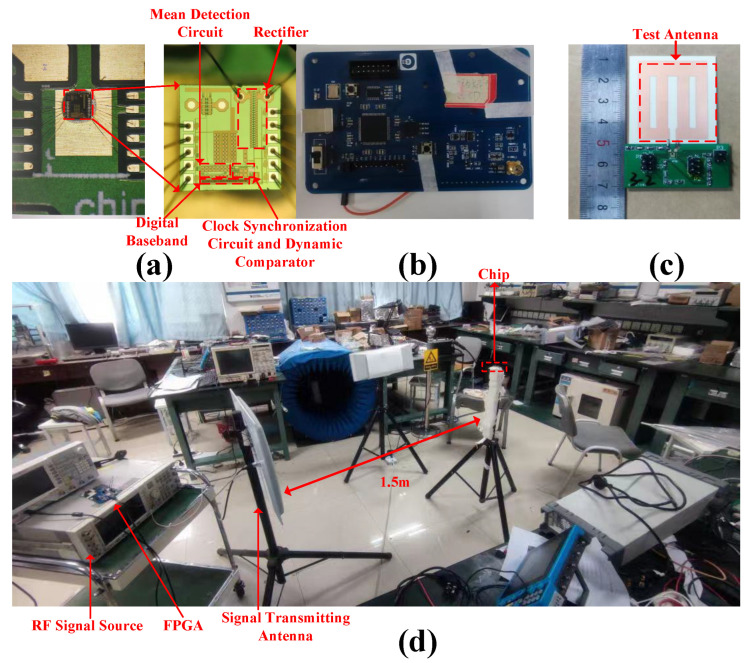
(**a**) Chip Bonding Diagram and Micrograph. (**b**) FPGA Platform Schematic. (**c**) Overall Test PCB Schematic. (**d**) Chip Testing Environment.

The wake-up receiver chip presented in this work was fabricated using a TSMC 0.18 µm CMOS process. The packaged die was mounted on a custom printed circuit board (PCB) using gold wire bonding technology. Figure 16a shows the chip micrograph and the bonding configuration diagram, where the chip pads are connected to PCB pads via gold wires to route signals from key nodes to external test equipment. The characterization was performed in an over-the-air (OTA) radiation setup [25].The chip was powered by a 0.5 V supply, provided by a high-precision source measurement unit. The test stimulus was generated by an FPGA (whose hardware platform is shown in Figure 16b, which produced a baseband modulated signal with a data rate of 2 kbps. This signal was then up-converted to a 922.5 MHz carrier frequency by an RF signal source and transmitted via a transmitting antenna. The receiver chip was connected to a custom antenna through the antenna interface on the PCB. The complete test platform architecture is illustrated in Figure 16c,d.

For the sensitivity measurement, an equivalent calculation method was employed due to laboratory condition constraints. With a fixed distance of 1.5 m between the transmitting antenna and the receiver chip, a spectrum analyzer measured the actual received power at the receiver antenna port as −11.5 dBm when the transmit power was set to 0 dBm, corresponding to a path loss of 23.5 dB. To emulate the −47 dBm received sensitivity condition, the output power of the RF signal source was set to −35.5 dBm. A total antenna gain of 12 dB was accounted for in this calculation to ensure the signal power arriving at the chip’s antenna input met the test requirement. A successful wake-up trigger generated by the subsequent circuitry under these conditions indicates that the chip sensitivity meets the −47 dBm specification.

### 4.2. Key Node Waveforms and Circuit Functionality Verification

**Figure 17:**
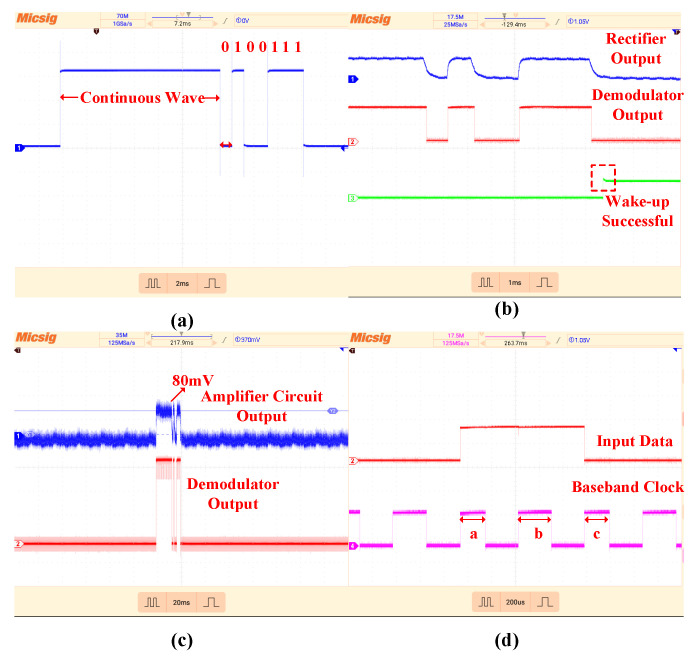
(**a**) FPGA Output Signal Waveform. (**b**) Output Waveforms at Key Nodes during Chip Testing. (**c**) Test Output Waveforms of the Amplification and Demodulation Circuits. (**d**) Test Verification of the Clock Synchronization Logic.

Figure 17a shows the modulated signal waveform output from the FPGA. Under the specified test conditions, the waveforms at key nodes of the chip are depicted in Figure 17b. Experimental results demonstrate that the rectifier output exhibits a well-defined envelope, the demodulation circuit functions correctly, and a valid wake-up command is successfully generated upon recognition of the preset wake-up code, indicating proper operation of the digital baseband. To further analyze the demodulation performance, the output waveform of the amplification circuit was examined, as shown in Figure 17c. The circuit provides a gain of approximately 3, amplifying the output signal peak to 80 mV, which aligns with simulation results. To verify the clock synchronization mechanism, Figure 17d illustrates the timing relationship between the demodulated output and the baseband clock. It can be observed that the pulse widths near the data transition edges (pulses aand c) are significantly narrower than other pulses. This indicates that the main clock was reset before completing a half-cycle, thereby truncating the corresponding baseband clock pulse, which is derived from the main clock through frequency division and delay. After reset, the baseband clock generates a rising edge at the mid-point of the bit period (pulse b), positioning it precisely at the optimal sampling point, which confirms the effectiveness of the clock synchronization logic.

Measurement results indicate that the chip consumes an average current of 0.61 µA at a 0.5 V supply voltage. The implemented wake-up receiver achieves a sensitivity of −47 dBm with a power consumption of only 305 nW, meeting the design targets for both ultra-low power consumption and high sensitivity.

Table 1 summarizes the key performance metrics of the wake-up receiver implemented in this work and provides a comparative analysis with existing state-of-the-art research.

## 5. Conclusions

Addressing the core demands of low power consumption and long-range communication for IoT sensor nodes, this paper presents an event-triggered, ultra-low-power wake-up receiver. The design employs a unique system architecture in which a nanowatt-level wake-up circuit continuously monitors the channel and activates the higher-power main receiver only after validating a legitimate wake-up command, thereby significantly reducing the overall system power consumption. Based on a custom co-design of the antenna and rectifier, the RF front-end achieves high-Q, low-power RF envelope detection. The adaptive mean-detection demodulator supports modulation depths as low as 30%, while the Barker code-based self-correlation logic enables robust wake-up detection with high interference and noise immunity. The data-edge-triggered synchronous clock reset circuit eliminates accumulated errors, increasing the tolerance to clock frequency deviation to over 16%. Operating with a 1 kHz data rate under a 0.5 V supply, the chip consumes only 305 nW of power and achieves a sensitivity of −47 dBm in the 920–925 MHz frequency band. This work also acknowledges several limitations, such as suboptimal circuit optimizations (e.g., asymmetric gain in the amplifier), untested metrics, including bit error rate (BER) and false wake-up rate, and susceptibility of the high-Q resonant system to environmental variations. These aspects will be the focus of our subsequent research.

## Figures and Tables

**Table 1:** Performance summary and comparison with other papers.

Parameters	[22]	[26]	[27]	[28]	[29]	This Work
Technology (nm)	90	65	-	180	130	180
Operating Voltage (V)	0.5	0.75	3.3	1.5	0.55	0.5
Carrier Frequency (MHz)	400	2400	868	2400	830	922
Power Consumption (nW)	$1.79\times 10^{3}$	$90\times 10^{3}$	$10.3\times 10^{3}$	$38.7\times 10^{3}$	64	305
Sensitivity (dBm)	−40	−24	−68.6	−39.9	−30.7	−47
Off-chip Matching Network	Yes	Yes	Yes	Yes	No	No

## Data Availability

The original contributions presented in this study are included in the article. Further inquiries can be directed to the corresponding author.
